# Associations between compulsory community treatment and continuity of care in a three year follow-up of the Oxford Community Treatment Order Trial (OCTET) cohort

**DOI:** 10.1186/s12888-017-1319-x

**Published:** 2017-04-28

**Authors:** Stephen Robert Puntis, Jorun Rugkåsa, Tom Burns

**Affiliations:** 1 Department of Psychiatry, University of Oxford, Warneford Hospital, Oxford, OX3 7JX UK; 2 Health Services Research Unit, Akershus University Hospital, Lørenskog, Norway

**Keywords:** Continuity of care, Outpatient compulsion, Community mental health, Psychosis

## Abstract

**Background:**

Most studies investigating the effectiveness of Community Treatment Orders (CTOs) use readmission to hospital as the primary outcome. Another aim of introducing CTOs was to improve continuity of care. Our study was a 3-year prospective follow-up which tested for associations between CTOs and continuity of care.

**Methods:**

Our study sample included 333 patients recruited to the Oxford Community Treatment Order Trial (OCTET). We collected data on continuity of care using eight previously operationalized measures. We analysed the association between CTOs and continuity of care in two ways. First, we tested the association between continuity of care and OCTET randomisation arm (CTO versus voluntary care via Section 17 leave). Second, we analysed continuity of care and CTO exposure independent of randomisation; using any exposure to CTO, number of days on CTO, and proportion of outpatient days on CTO as outcomes.

**Results:**

197 (61%) patients were made subject to CTO during the 36-month follow-up. Randomisation to CTO arm was significantly associated with having a higher proportion of clinical documents copied to the user but no other measures of continuity. Having a higher proportion of outpatient days on CTO (irrespective of randomisation) was associated with fewer 60 day breaks without community contact. A sensitivity analysis found that any exposure to CTO and a higher proportion of outpatient days on CTO were associated with fewer days between community mental health team contacts and 60 day breaks without contact.

**Conclusion:**

We found some evidence of an association between CTO use and better engagement with the community team in terms of increased contact and fewer breaks in care. Those with CTO experience had a higher number of inpatient admissions which may have acted as a mediator of this association. We found limited evidence for an association between CTO use and other measures of continuity of care.

## Background

Compulsory treatment in mental health care was traditionally limited to psychiatric inpatient settings. However, over the last 30 years compulsory treatment in the community has been introduced in over 70 jurisdictions worldwide [1]. Community Treatment Orders (CTOs) were introduced in England and Wales in the 2007 amendments to the Mental Health Act. Clinicians’ adoption of the use of CTO has been rapid, despite the controversy surrounding the evidence for its effectiveness [2].

The intention behind amending the Mental Health Act to include CTOs was “to allow suitable patients to be safely treated in the community rather than under detention in hospital, and to provide a way to help prevent relapse…” [3]. This description in the Mental Health Act Code Of Practice hints at the multiple aims of CTOs, including to improve patient functioning, to reduce hospital use, to prevent relapse, and to reduce risk. The primary outcome used in trials investigating the effectiveness of CTOs has been rates of hospital admission. However, a range of other outcomes have been tested to reflect these multiple aims including medication adherence, health service use, reduction in violence, functioning, and quality of life [4]. Improving continuity of care for patients was also an aim when CTOs were introduced in England and Wales. Patients who are made subject to CTOs may be those for whom good continuity is most difficult to achieve and it was argued that CTOs would improve services’ ability to provide continuity through facilitating contact and compliance with treatment [5, 6]. However, critics of CTOs have argued the opposite; CTOs may cause patients to disengage from mental health services, resulting in poor long-term continuity [7].

Studies examining the effect of CTOs on continuity of care have mostly been limited to a narrow set of outcomes such as frequency of contact with community services, or uptake in the use of community services. Most current evidence suggests that patients are seen by their community team more often on CTO, and have a higher uptake of community services such as assertive community treatment, and reduction in the use of crisis team [8–13]. In some cases these results may be related to the regional variation in CTO legislation. For example, in New York, enhanced multidisciplinary community outreach services cannot be initiated without CTOs [14]. Whilst the maintenance of clinical contact and medication adherence are critical aspects in the decision to use CTOs (and are the most frequently used discretionary CTO conditions specified in England and Wales), the orders are likely to indirectly influence other dimensions of continuity of care. For instance, discretionary conditions of CTOs can include residency requirements (such as remaining in social housing) and engaging in psychological and social rehabilitation interventions [15], and the use of CTOs may influence case management and care planning [16]. In our present study, we investigate the association between CTO and eight multidimentional markers of continuity of care.

Continuity of care is not a simple concept. It can be described as a process of delivering care over time which is coordinated and appropriate to a patient’s needs [17]. There is consensus that continuity of care is a multidimensional construct. Freeman and colleagues’ eight-dimensional definition, comprising experienced, flexible, cross-boundary, information, longitudinal, relational, long-term, and contextual continuity is a common example of how this multidimensionality has been characterised [18]. The Experiences of Continuity of Care and Health and Social Outcomes study (ECHO) operationalised Freeman and colleagues conceptualisation into 20 measures so that data on continuity could be reliably collected from medical records and patient interview [19]. In this study we used an adapted eight-component version of the ECHO measure for which data can be collected solely from medical records.

There is little data in the United Kingdom (UK) on the association between CTO use and continuity of care. We therefore investigated whether CTOs are associated with a wider range of continuity of care measures by prospectively following patients recruited to the Oxford Community Treatment Order Evaluation Trial (OCTET) for 36 months [20]. OCTET was a multi-centre randomised control trial (RCT) in England that randomised patients to either Community Treatment Order (CTO) or to voluntary treatment via short-term Section 17 leave (further information on the randomisation in OCTET can be found elsewhere) [20–23]. The present analysis had two aims. First, we aimed to determine whether being subject to CTO is associated with improved continuity of care in the community. Second, we aimed to explore the relationship between the duration of CTO and continuity of care, as findings from previous studies suggest that CTOs need an extended time to ‘bed in’ to affect outcomes [24, 25].

We analysed the OCTET data using two different methods. In the first analysis we utilised the strength of the randomised design from the OCTET trial. In the second we analysed the entire OCTET cohort, irrespective of randomisation in order to account for crossovers, as clinicians were not asked to maintain patients on the intervention they were randomised to beyond the initial 12 month RCT follow-up.

## Methods

### Sample and data collection

Our sample was 333 of the 336 patients randomised to OCTET. Of the three patients excluded, one patient withdrew during the trial and two were found to be ineligible directly after randomisation. Patients eligible for the trial were those aged 18 to 65 years with a diagnosis of psychosis, currently detained in an inpatient hospital under Section 3 or Section 37 of the Mental Health Act (MHA). They had to be candidates for CTO at discharge, and able to give informed consent to take part in research. Patients were followed for 36 months from randomisation. For the present study we excluded patients who were inpatients throughout the 36 months as there would be no community service use to measure. Data at baseline were collected using a combination of medical records and patient interview, and the follow-up data reported here were collected entirely from medical records. Full recruitment and data collection methods are described elsewhere [26]. Ethical approval was granted by the Staffordshire National Health Service (NHS) Research Ethics Committee (reference 08/H1204/131).

### Measures

#### Baseline measurements

We collected data on age, gender, ethnicity, country of birth, years of education, marital status, diagnosis, duration of illness, and number of past psychiatric hospitalisations. We measured severity of symptoms with the Brief Psychiatric Rating Scale (BPRS) [27].

#### Outcome measurements

We used measures of continuity of care operationalised by the Experiences of Continuity of Care and Health and Social Outcomes study [19]. We applied eight of their 20 components in our study (Table 1). The eight components included were:

**Table 1:** ECHO continuity of care factor structure and components

Factor	Factor name	Description	Components *(later omitted)* ^*a*^
1	Experience and relationships	High experienced continuity, good therapeutic relationship, a greater proportion of needs met and not having a user-rated break in care.	• *CONTINU-UM* ^*b*^ • *STAR total score – any professional* ^*c*^ • *Proportion of needs met* • *Any user-rated breaks in care*
2	Regularity	Being seen more frequently by staff from fewer different non-medical disciplines.	• Average gap between face-to-face contacts • Gaps of 2 months or more • Non-medical input spread
3	Meeting needs	High level of need, high number of met needs and CPA copied to GP and user.	• *CAN total level of needs* ^*d*^ • *CAN number of met needs* ^*d*^ • *CPA copied to GP and user* ^*e*^
4	Consolidation	Having contact with fewer different agencies and not seeing primary care professionals.	• *Number of agencies used in previous year* • *Contacts with primary care professionals*
5	Managed transitions	Having no transition, having a transition and it was documented. Having a transition that was undocumented	• *Had a transition?* • Documented transition
6	Care Coordination	Having a designated care coordinator, having no psychiatrist or more than two and fewer needs met by informal carers.	• Designated care coordinators • Designated psychiatrists • *CAN total level of needs met by informal carers* ^*d*^
7	Supported Living	Living in supported accommodation, attending day care and having more letters copied to the user.	• Supported accommodation • *Attendance at day centre or hospital* • Proportion of letters sent by CMHT or copied to user

^a^Items in italics were not collected in our study, for reasons given in text

^b^CONTINU-UMis the ECHO study Continuity of Care User Measure

^c^STAR is the Scale to Assess Therapeutic Relationships in Community Mental Health Care

^d^CAN is the Camberwell Assessment of Need

^e^CPA is the Care Program Approach

*Average gap between face-to-face contacts*: the mean number of days between each face-to-face contact for a patient and any member of their community team. It is an operationalisation of the continuing relationship between patient and community team.

*Gaps of two months or more*: the number of instances when 60 days or more passed between a successful face-to-face contact with the community team and the next such contact. It measures breaks in care, which may indicate a breakdown in the relationship between patient and team.

*Number of professions*: the sum of different non-medical professions the patient has been in contact with. Each profession is only counted once; if several members of the same profession saw the same patient they would count as one. It is a reconceptualization of the ECHO component *non-medical input spread* [28]. Seeing a wider range of professions represents more comprehensive, wrap-round care.

*Number of designated care coordinators*: the total number of care coordinators the patient was assigned to during the follow-up period. A change in care coordinator (also known as a keyworker) indicates a discontinuity in the relationship between patient and community team.

*Number of designated psychiatrists*: the total number of consultant psychiatrists responsible for the patient’s care during the follow-up period. A change in psychiatrist indicates a discontinuity in the relationship between patient and community team.

*Supported living*: measured as whether the patient was discharged to supported accommodation from their index hospitalisation. It is the ECHO operationalisation of Freeman and colleagues’ *contextual continuity*, the social context reflected in a patient’s living situation and daily activities.

*Documented transitions:* a binary measure of whether the patient had any transition documented throughout the follow-up that is supported by a recorded referral letter or email with information about the patient. It represents Freeman and colleagues’ *continuity of information*; the information transfer which follows the user.

*Proportion of documents copied to user*: the proportion of all documents containing information about the patient’s care sent by the Community Mental Health Team (CMHT) which are either addressed to the patient or the patient has been copied in to. It has three categories: having 0% of documents copied to the patient; having 1–50% of documents copied to the patient; having 51-100% of documents copied to the patient. It is an operationalisation of continuity of information between patient and community team.

The remaining 12 ECHO components were excluded for the following reasons: Two components were excluded as they required patient interview (*CONTINU-UM*; *Any user-rated breaks in care*). Four were excluded after piloting the data collection process, as they could not be reliably ascertained from medical records (C*ontacts with primary care professionals*; *Care Programme Approach (CPA) copied to general practitioner (GP) and user*; *Number of agencies used in previous year*; *Attendance at day centre or hospital*). *Had a transition* was excluded as all patients in this study by default had at least one transition as they were all discharged from hospital after being recruited. The *Scale To Assess the therapeutic Relationship (STAR)* was excluded as it is a measure of the therapeutic relationship and not continuity of care. The *Camberwell Assessment of Needs (CAN) number of met needs*; *CAN total level of needs met by informal carers*; *CAN total level of needs*, and; the *Proportion of needs met* were excluded because we consider them outcomes of, rather than the process of, continuity of care.

### Outcomes

CTO experience was measured in four ways to account for both changes in patients’ CTO status and the duration of CTO.

*Randomised to CTO* compared patients randomised in OCTET to either CTO or voluntary treatment via Section 17 leave (which allows patients who remain subject to detention for treatment under section during leave in the community). A full description and explanation of randomisation arms can be found elsewhere [20, 21].

*Subject to CTO* was a binary measure of whether a patient had been subject to CTO at any point during the 36-month follow-up, **irrespective of initial randomisation**. This measure accounted for changes in patients’ legal status post-randomisation.

*Number of days on CTO* measured the total number of days subject to CTO until end of follow-up, irrespective of randomisation. Where applicable, multiple periods of CTO were summed to yield a total number of days. Those never subject to CTO were recorded as having zero days. We used this measure to investigate duration of CTO on continuity outcomes.

*Proportion of outpatient days on CTO* was the number of CTO days as a proportion of the total number of days as an outpatient, irrespective of randomisation. We used this measure to account for variations in the length of inpatient episodes.

### Analysis

We calculated descriptive statistics using measures of central tendency and variability. We compared patients who had any CTO experience against those who did not for number of readmissions, number of days in hospital, and number of days under compulsion using a t-test (t) or Mann-Whitney U test (U) for non-parametric data.

The appropriate regression models were selected for testing associations between the four measures of compulsion and the eight outcomes, 32 comparisons in all. We fitted linear regression models to test for associations between the four measures of CTO and average gap between face-to-face contacts, reported in beta values (B) with 95% confidence intervals (95%CI). We aimed to use Poisson regression models for each of gaps of 2 months or more, number of professions, designated care coordinators, and designated psychiatrists. If the outcome suffered from overdispersion, we used a negative binomial model instead. These outcomes are reported in Incidence Rate Ratios (IRRs) with 95%CIs. We used logistic regression to test for associations between CTO measures and supported living and documented transitions, and report them as Odds Ratios (ORs) with 95%CIs. Log multinomial regression was used to test for associations between CTO measures and proportion of documents sent by the CMHT which were copied to the user. It is reported as relative risk (RRs) with 95%CIs. We adjusted all analyses for age, gender, ethnicity, and BPRS score.

To correct for multiple testing we used a Bonferroni correction, α/n, where α = 0.05 and *n* = 32. Therefore a significance level of equal to or less than 0.001 was required in order to reject the null hypothesis.

We conducted two sensitivity analyses. The first was conducted to test for associations between the duration on CTO and continuity of care only for the patients who were made subject to CTO. The second sensitivity analysis reran the analyses that included the whole sample but excluded outliers identified in scatterplots during data cleaning.

## Results

### Participants

Ten of the 333 patients were excluded: five were inpatients throughout the follow-up; data for four could not be collected; and one withdrew consent. The final sample therefore included 323 patients. There were 20 deaths: six from suicide; one accidental death from a drug-overdose; and 13 from natural causes. Three patients moved abroad during the study and 23 patients were discharged from community mental health services. Five of these 23 patients were later re-referred and collection of their data recommenced from the date of referral. Data for patients who died or were discharged were censored at the relevant time point.

### Sample baseline characteristics

Patients’ mean age was 39.59 (SD = 11.39) and 218 (67.5%) were male. Most had been diagnosed with schizophrenia (*n* = 275, 85.1%). The mean number of previous hospitalisations was 6.7 (SD = 6) and patients had first been diagnosed a mean of 14.42 years (SD = 10.45) previously. 196 were White (60.7%). Only 28 patients were married or cohabitating (8.7%) and two patients (0.6%) were in regular employment.

### Differences in clinical outcomes between those subject to CTO and those not

Table 2 presents demographic characteristics and clinical outcomes between patients made subject to a CTO and those who had not been made subject to a CTO, independent of randomisation.

**Table 2:** Demographic characteristics and clinical and continuity outcomes between patients made subject to a CTO and those who had not been made subject to CTO

		CTO = 197		Non-CTO = 126	
	N	N Mean Median	% SD IQR	N Mean Median	% SD IQR
Mean age, *years* (SD)	323	39.59	11.24	39.48	11.68
Gender, male (%)	323	134	68%	84	66.8%
Ethnicity, *White* (%)	323	114	57.9%	82	65.1%
Mean duration of illness, *years* (SD)	313	14.41	10.01	14.42	11.17
Mean BPRS total score at baseline (SD)	302	38.62	10.87	38.92	12.15
Readmitted, *yes* (%)	323	131	66.5%	75	59.5%
Number of readmissions	323	1.63	1.83	1.17	1.55
Median number of days in hospital over 36 months[IQR]	323	116.00	34.50, 228	83.50	25.75, 300
Median number of days in hospital from 1st readmission [IQR]	323	74	0, 177.5	18.50	0, 139
Median number of days under any compulsion^a^ [IQR]	323	605	342.50, 1088	120	38, 248
Number of days between face-to face-contacts	321	8.86	5.69, 14.11	11.25	6.53, 18.83
Number of 60-day gaps,	321				
No gaps		117	59.7%	64	50.8%
1–2 gap		60	30.6%	36	28.6%
3 or more gaps		19	9.7%	26	20.6%
Number of different non-medical professional	319	5.76	0.895	5.38	0.72
Designated care coordinators	319	2.40	1.28	2.15	1.23
Designated psychiatrists	319	3.89	3.04	3.28	2.289
Discharged to supported accommodation, yes	321	47	23.9%	24	19.2%
Any referral documented, yes	314	110	55.8%	66	54.1%
Proportion of letter copied to user	323				
0%		53	26.9%	38	30.2%
1–50%		79	40.1%	53	42.1%
51–100%		65	33%	35	27.8%

^a^Includes both inpatient and outpatient compulsion

One hundred ninety seven (61%) patients had been subject to a CTO during the study, with 145 (44.9%) on CTO for more than 180 days. Patients who were made subject to CTO remained on it for a median of 353 days (IQR = 180, 725.50) days, with a range between 7 and 1088 days. The number of readmissions was significantly greater for those in the CTO experience group (*n* = 323, U = 2.45, *p* = 0.014). The total number of days under compulsion (defined as being on any MHA section) was also significantly greater for the CTO group (*n* = 323, U = 11.6, *p* < 0.001).

### Associations between CTO status and continuity of care

Only one continuity of care outcome was associated with randomisation arm at the Bonferroni adjusted significance level (Table 3). *CTO randomisation* was significantly associated with an increased chance of having a higher proportion of documents copied to the user (RR = 2.768, 95%CI = 1.490, 5.140, *p* = 0.001). There were no significant associations between either *Subject to CTO* or *Number of days on CTO* and the continuity of care outcomes (Table 3). *Higher proportion of outpatient days on CTO* was associated with a reduced incidence of 60 day gaps (IRR = 0.423, 95%CI = 0.257, 0.696, *p* < 0.001). There were no other significant associations between the proportion of outpatient days on CTO and continuity of care.

**Table 3:** Multivariate associations between CTO experience and continuity of care variables, controlling for age, gender, ethnicity and symptom severity

Measure	Randomised to CTO		Subject to CTO		Number of days on CTO		Proportion of outpatient days on CTO	
	Beta/OR/IRR/RR and 95% CI	*p*-value	Beta/OR/IRR/RR and 95% CI	*p*-value	Beta/OR/IRR/RR and 95% CI	*p*-value	Beta/OR/IRR/RR and 95% CI	*p*-value
Average gap between face-to-face contacts^a^	1.402 (−4.981, 2.176)	0.441	−5.090 (−8.71, −1.47)	0.006	−0.006 (−0.011, −0.001)	0.020	−6.954 (−11.839, −2.069)	0.022
Number of 60 day gaps without contact^b^	0.919 (0.660, 1.280)	0.617	0.615 (0.440, 0.859)	0.004	0.999 (0.999, 1.000)	0.003	**0.423 (0.257, 0.696)**	**0.001**
Number of different mental health professions seen, *per patient* ^*b*^	1.002 (0.781, 1.281)	0.988	1.075 (0.836, 1.383)	0.574	1.000 (1, 1)	0.596	1.088 (0.773, 1.531)	0.630
Number of care coordinators, *per patient* ^*b*^	1.069 (0.813, 1.404)	0.634	1.113 (0.840, 1.474)	0.456	1.000 (1, 1)	0.992	1.076 (0.738, 1.570)	0.702
Number of psychiatrists, *per patient* ^b^	1.007 (0.780, 1.301)	0.956	1.203 (0.923, 1.567)	0.171	1.000 (1, 1)	0.844	1.203 (0.842, 1.720)	0.310
Discharged from index admission to support accommodation, *yes* ^c^	0.930 (0.533, 1.621)	0.797	1.361 (0.759, 2.439)	0.301	1.000 (1.000, 1.001)	0.920	1.740 (0.831, 3.644)	0.142
Any referral documented, *yes* ^*c*^	1.063 (0.661, 1.707)	0.802	1.242 (0.764, 2.019)	0.383	1.000 (0.999, 1.001)	0.910	1.123 (0.580, 2.174)	0.730
Proportion of documents copied to user (0%)^d^								
*1–50%*	1.809 (1.017, 3.218)	0.044	1.215 (0.690, 2.140)	0.500	1.000 (0.999, 1.001)	0.971	1.036 (0.359, 2.988)	0.948
*51–100%*	**2.768 (1.490, 5.140)**	**<0.001**	1.352 (0.732, 2.496)	0.355	1.000 (0.999, 1.001)	0.592	1.232 (0.412, 3.688)	0.709

Items in bold are significant at the level shown in the table

^a^Outcome analysed using linear regression

^b^Outcome analysed using negative binomial regression

^c^Outcome analysed using logistic regression

^d^Outcome analysed using log multinomial regression

### Sensitivity analyses

#### Only including patients subject to CTO

To test for associations between duration of CTO and continuity of care we reran the analyses excluding patients who had not been made subject to CTO. Neither the total number of days on a CTO nor the proportion of outpatient days on CTO was significantly associated with any measure of continuity of care.

#### Removal of outliers

There were three patients who were identified as outliers by plotting the average gap between face-to-face contacts against CTO status, number of days on CTO, and proportion of outpatient days on CTO. On excluding these patients, being *subject to CTO* was significantly associated with a lower average gap between face-to-face contacts (B = −4.564, 95% CI = −6.978, −2.149, *p* < 0.001). The *proportion of outpatient days on CTO* was also significantly associated with the average gap between face-to-face contacts (B = −6.044, 95%CI = −9.315, −2.774, *p* < 0.001). The *number of days on CTO* was not significantly associated with average gap between face-to-face contacts (B = −0.006, 95%CI -0.009, −0.002, *p* = 0.002).

One patient was identified as an outlier by plotting the *number of days on CTO* or the *proportion of outpatient days on CTO* and the number of 60 day gaps. Excluding this outlier resulted in a significant association, with more days on a CTO associated with fewer 60 day gaps (IRR = 0.999, 95%CI = 0.998, 1.000, *p* < 0.001). There was also a significant association between a higher *proportion of outpatient days on CTO* and fewer 60 day gaps (IRR = 0.361, 95%CI = 0.215, 0.607, *p* < 0.001).

One patient was identified as an outlier by plotting the *number of days on CTO* or the *proportion of outpatient days on CTO* and the number of professionals seen. Neither the *number of days on CTO* nor the *proportion of outpatient days on CTO* was significantly associated with number of professionals seen when excluding this participant. No other outliers were identified for any of the other measures of continuity of care.

## Discussion

In this study we tested for associations between outpatient compulsion and continuity of care over 36 months, using four different measures of CTO exposure. The first tested the original OCTET randomisation trial arms, the second those subjected to a CTO at any point during the follow-up irrespective of randomisation, compared to no CTO. The third tested the total number of days subject to CTO, and the fourth the proportion of outpatient days subject to CTO, also both independent of randomisation.

We found little evidence of any effect of CTO on the eight measures of continuity of care using the original OCTET randomisation. Patients randomised to CTO had a higher proportion of clinical documents copied to them. No other measure of CTO experience was associated with continuity of care.

Examining CTO experience in the entire cohort produced a slightly different picture. In the main analysis those with a higher proportion of outpatient days on CTO had fewer gaps in contact with their CMHT. A sensitivity analysis, excluding outliers in the data, found that patients made subject to CTO had fewer days between community contacts and that more days on CTO were associated with fewer 60 day breaks in contact. Having a higher proportion of outpatient days on CTO was also associated with fewer days between community contacts and fewer 60 day breaks in care when outliers were excluded. No other measures were associated with exposure to CTO.

For patients on a CTO, the duration of time on CTO (measured by number of days on a CTO and proportion of outpatient days on a CTO) was not associated with any measures of continuity of care.

### The association between CTO and contact with the community mental health team

There was concern when CTOs were introduced that they would drive patients away from services [29]. We found no evidence of this. Patients who were made subject to CTO had more frequent contact and fewer gaps in contact than those without CTO experience.

Does this mean that CTOs improve contact with community services? Improving engagement with services is an explicit purpose of CTOs. It is also, along with medication adherence, the most frequent discretionary condition specified in CTO paperwork [15, 30]. Previous research in countries other than the UK has identified an increase in engagement for patients on CTO [10–13]. The participants in this study, both those with and without CTO experience, had what might be considered remarkably persistent clinical follow-up. Those with experience of CTO had approximately eight and a half more contacts a year than the non-CTO group, a difference between a mean of 32 and 41 contacts a year. In both groups more than half the patients did not have a single 60 day gap in their care.

However, the use of CTO might not have been the only factor which drove this increased frequency of contact. The results from our two methods of analysing this cohort are conflicting and both of our methods have limitations. The randomised groups suffered from a large number of crossovers, with 43.2% of the non-CTO arm made subject to CTO during the 3 year follow-up, whilst analysing by exposure to CTO introduced selection bias. Those with any exposure to CTO (independent of randomisation) had significantly more readmissions than those without CTO exposure. Patients who are admitted to hospital often have increases in community follow-up prior to admission and following discharge and the associations we found may have been driven by this different clinical pathway. A previous study of ours found that contact frequency was significantly associated with admissions [28]. Furthermore, the frequency of contact between a patient and community team is also likely to be influenced by a number of factors including severity of illness, perceived need, and availability of resources. It is also possible that following an admission, patients were placed on a CTO and this drove the increase in contact that we measured.

### Continuity of care in community mental health

The only other measure of continuity of care associated with CTO use was an increase in the proportion of clinical documents copied to patients. From our inspection of these letters, many of them were to the patient or other agencies with information specifically about the patient’s CTO. It is unclear whether this increase in the numbers of documents sent for those on CTO and the proportional increase in including patients in this correspondence represents a genuine improvement in information continuity (by better informing patients’ about their treatment), a reflection of the increased administration of using CTOs, or both. CTOs did not seem to improve continuity of care in other ways, such as with access to supported housing, which has been found in previous studies [9]. This may reflect the differences in CTO legislation in different countries. Another reason that CTOs appear not to be associated with measures of continuity of care other than frequency of contact may reflect UK patient and clinician understanding of their purpose. Qualitative interviews with patients, clinicians, and carers have found that all three groups considered the primary purpose of CTOs to be the enforcement of adherence to treatment [31].

Finally, there are no widely accepted measures of continuity of care. We aimed to adhere to the ECHO measures rather than create new measures to avoid the current lack of comparability of measures in continuity of care research [32]. However, some had to be adapted because we only collected data from medical records. This modification of the instrument to only collect data from medical records is a limitation of this study. Measures of patients’ perception of their continuity of care when subject to CTO may differ from the service measures which we used.

## Conclusion

In conclusion, we found an association between CTO use and engagement with services. We were however unable to determine whether this was due to the effect of the CTO, or due to a mediating effect such as increased hospitalisation or illness severity. We also found a strong association between CTO use and an increased number of documents copied to the patient.

The evidence of whether CTOs keep patients out of hospital is controversial. We would argue a modest improvement in the frequency of community contact combined with what we consider limited evidence for the effectiveness of CTOs in reducing hospitalisation and other outcomes may not justify their use.

## Abbreviations

BPRS: Brief Psychiatric Rating Scale
CAN: Camberwell Assessment of Needs
CMHT: Community Mental Health Team
CPA: Care Programme Approach
CTO: Community Treatment Order
ECHO: Experiences of Continuity of Care and Health and Social Outcomes Study
GP: general practitioner
IRR: Incidence Rate Ratio
MHA: Mental Health Act
NHS: National Health Service
OCTET: Oxford Community Treatment Order Evaluation Trial
OR: Odds Ratio
RCT: Randomised Controlled Trial
RR: Relative Risk
STAR: Scale to Assess the Therapeutic Relationship
UK: United Kingdom
